# USP7 promotes cervical cancer progression by stabilizing MTDH expression through deubiquitination

**DOI:** 10.1007/s00432-024-05710-9

**Published:** 2024-04-16

**Authors:** Na Wang, Jing Xu, Yujing Wang, Xuejiao Zhang, Hongzhen Zhang

**Affiliations:** 1 Department of Gynecology, The First Hospital of Hebei Medical University, No. 89, Donggang Road, Yuhua District, Shijiazhuang City, 050031 Hebei Province China; 2 Department of Obstetrics, The First Hospital of Hebei Medical University, Shijiazhuang, Hebei China

**Keywords:** Deubiquitination, USP7, MTDH, Macrophage, M2 polarization, Apoptosis, Immune escape

## Abstract

**Background:**

Metadherin (MTDH) and ubiquitin specific protease 7 (USP7) have been identified to involve in the tumorigenesis of cervical cancer (CC). USP7 is one of the deubiquitinating enzymes. Here, this study aimed to explore whether USP7 affected CC progression via interacting with MTDH and regulating its stability via deubiquitination.

**Methods:**

qRT-PCR and western blotting assays detected the levels of genes and proteins. Functional analysis was conducted using 5-ethynyl-2’-deoxyuridine (EdU), flow cytometry, transwell, and tube formation assays, respectively. Proteins between USP7 and MTDH were identified by co-immunoprecipitation assay. A mouse xenograft model was established for in vivo analysis.

**Results:**

MTDH was highly expressed in CC tissues and cells, silencing of MTDH suppressed CC cell proliferation, migration, invasion, angiogenesis, and macrophage M2 polarization. Mechanistically, USP7 directly bound to MTDH, and maintained its stability by removing ubiquitination on MTDH. CC tissues and cells showed high USP7 expression, and USP7 knockdown also inhibited CC cell proliferation, migration, invasion, angiogenesis and macrophage M2 polarization, and these effects mediated by USP7 knockdown were reversed by MTDH overexpression. Moreover, USP7 knockdown impeded CC growth in vivo by regulating MTDH.

**Conclusion:**

Collectively, USP7 promoted CC cell proliferation, migration, invasion, angiogenesis, and macrophage M2 polarization in vitro, as well as tumor growth in vivo by regulating MTDH.

## Introduction

Cervical cancer (CC) is a common malignant tumor in the female reproductive system, with a high incidence and mortality rate (Sung et al. 2021). Although the basic popularization of CC screening in clinical work, most patients are still diagnosed at the late stage, posing a fatal threat to the survival rate of patients (Allahqoli et al. 2022; Mattern et al. 2022). In addition, over one-third of CC patients are still prone to local recurrence, lymph node metastasis or distant metastasis after first-stage treatment, which is one of the main reasons for the poor prognosis of CC patients (Cohen et al. 2019). Thus, further clarifications on the pathogenesis of CC are essential for developing novel molecular targets to prolong the survival rate of these patients.

Metadherin (MTDH, also known as LYRIC and AEG-1) is a well-known oncogene that allows cancer cells to adhere closely to blood vessels to metastasis (Hu et al. 2009), moreover, MTDH protein promotes angiogenesis and confers chemoresistance (Liu et al. 2009; Yoo et al. 2009). MTDH has been identified to be increased in many cancers, and plays a key role in cancer progression (Shi and Wang 2015). For example, MTDH was tightly related to advanced tumor grade and stages in patients with colorectal cancer (CRC) (Sultan et al. 2021), and could enhance CRC cell proliferation, anaerobic glycolysis and stemness via activating NF-ĸB pathway (El-Ashmawy et al. 2019). DOT1L could promote MTDH-evoked proliferation, angiogenesis, and invasion in breast cancer cells by the epigenetic regulation through NF-κB-HIF1α axis (Neeli et al. 2023). MTDH-induced autophagy activation in gastric cancer via AMPK/ATG5 signaling pathway and then enhanced 5-FU resistance in cancer cells (Pei et al. 2018). In CC, high expression of MTDH predicted shorter survival rates, and was closely related to lymph node metastasis and vascular invasion (Huang et al. 2013; Yu et al. 2015). Knockdown of MTDH could enhance radiosensitivity and chemosensitivity by inducing CC cell apoptosis and the arrest of cell proliferation and autophagy (Zhao et al. 2012; Zhang et al. 2013). Shi et al*.* found that HSF1 could elevate MTDH level to accelerate cancer cell growth and mobility (Shi et al. 2021). Thus, targeting the inhibition of MTDH may be a promising therapeutic method for CC.

Currently, increasing evidence has hinted the significance of abnormal epigenetic regulation of gene function in cancer genesis and progression (Miranda Furtado et al. 2019). According to the prediction of PrePP website (https://honiglab.c2b2.columbia.edu/hfpd/cgi-bin/index_jmol.cgi?view=analysis_jmol&uniprot=MTDH), ubiquitin specific protease 7 (USP7), the sole-predicted deubiquitylating enzyme, may interact with MTDH protein. Ubiquitination is a common post-translational modification that plays a significant role in multiple biological processes, importantly, the dysregulation of ubiquitination and deubiquitination is conducive to disease progression, including cancer (Sun et al. 2020; Pergolizzi et al. 2019; Cockram et al. 2021). As one of the deubiquitinating enzymes (DUB), USP7 can erase ubiquitin and suppress the degradation of substrate protein (Wang et al. 2016). Moreover, the previous study showed that USP7 was elevated in GCC, and promoted CC cell growth and suppressed the sensitivity of cells to genotoxic insults by stabilizing MDC1 via deubiquitination (Su et al. 2018). Li et al*.* showed that USP7 elevated EZH2 by TIMP2, and then promoted the growth, mobility, and immune escape in CC cell (Li et al. 2022). Here, we speculated that USP7 might deubiquitinate and stabilize MTDH to regulate CC progression.

Herein, this work investigated the action of USP7 and MTDH in CC cell oncogenic phenotypes and immune escape, and further probed whether USP7 affected CC progression via stabilizing MTDH through the deubiquitination.

## Materials and methods

### Samples collection

A total of 53 CC patients were included in this study. The tumor tissues and matched normal tissues were collected during surgery, and pathologically diagnosed. All the subjects did not accept preoperative treatments. The tissues were reserved at – 80 °C. This study was allowed by the Ethics Committee of the First Hospital of Hebei Medical University. All subjects have signed the written informed consent.

### Cell culture

Human CC cell lines (SiHa and HeLa) and normal End1/E6E7 cells were purchased from Procell (Wuhan, China). End1/E6E7 cells were cultured in DMEM (Lonza, Basel, Switzerland), and CC cell lines were cultured in MEM medium (Lonza). All media were added with 10% FBS and 1% penicillin/streptomycin (Lonza), and maintained in 5% CO_2_ at 37 °C.

### Quantitative real-time PCR (qRT-PCR)

Total RNA was extracted using the TRIzol reagent (Takara, Dalian, China), then cDNA was synthesized with HiScript III RT SuperMix (Vazyme, Nanjing, China), followed by qRT-PCR analysis with the SYBR Green Taq Mix (Takara) and specific primers (Table 1). The relative expression was calculated by the 2^–ΔΔCT^ method with GAPDH and U6 as internal references.

**Table 1:** Primers sequences used for qRT-PCR

Name	Primers for qRT-PCR (5ʹ-3ʹ)
MTDH	
Forward	GCACTAGTGATCCAGCCGAA
Reverse	TAGTATTGGCGGCACTTGGG
USP7	
Forward	CCGAGGACATGGAGATGGAAG
Reverse	AGGGCCACATTCCCATTGAT
IL-10	
Forward	TCTCCGAGATGCCTTCAGCA
Reverse	TCACATGCGCCTTGATGTCT
TGFβ1	
Forward	TGATGTCACCGGAGTTGTGC
Reverse	GTGAACCCGTTGATGTCCACT
GAPDH	
Forward	ATCACTGCCACCCAGAAGAC
Reverse	CCGTTCAGCTCAGGGATGAC

### Western blotting

Total proteins were isolated using RIPA lysis buffer containing protease and phosphatase inhibitor cocktail (Beyotime, Beijing, China). The concentration of proteins was determined using a BCA assay, and then separated by 8% SDS-PAGE gel, followed by shifting to nitrocellulose membranes. Then MTDH (ab124789, 1:10,000), USP7 (1:2000, ab108931) and GAPDH (ab8245, 1:1000) (Abcam, Cambridge, MA, USA) were used to incubate with the membranes at 4 °C for 12 h, which were then probed with secondary antibodies (1:5000) (Abcam) for 2 h at 37 °C. Finally, protein blots were assayed using the ECL Reagent Kit (Invitrogen, Carlsbad, CA, USA).

### Cell transfection

The small interference RNA (siRNA) targeting MTDH or USP7 (si-MTDH or si-USP7) or the short hairpin RNA (shRNA) targeting USP7 (sh-USP7) were designed by GenePharma with nontargeted siRNAs or shRNAs as the negative control (si-NC or sh-NC). MTDH-overexpression plasmids (MTDH) were established by cloning full-length of MTDH into pcDNA3.1 plasmids (GenePharma), and the empty pcDNA3.1 plasmids were used as the negative control (pcDNA). The transient transfection was performed using the Lipofectamine 3000 (Invitrogen).

### 5-Ethynyl-2ʹ-deoxyuridine (EdU) assay

SiHa and HeLa cells were reacted with 50 μM EdU solution (RiboBio, Guangdong, China) for 3 h. After fixation and permeabilization, cells were dyed with 1 × Apollo^®^ reaction cocktail for 30 min. Cell nuclei were stained with 1 µg/mL DAPI for 10 min. Lastly, EdU-positive cells were assessed using a fluorescent microscope.

### Flow cytometry

After being re-suspended in 500 µL binding buffer, SiHa and HeLa cells were double-dyed with 10 µL Annexin V-FITC and 10 µL propidium iodide (PI) (BD Biosciences) for 15 min avoiding light. Finally, apoptotic cells were counted using the flow cytometer.

### Transwell assay

Transwell plates (8-µm pore size; Corning, Inc., Corning, NY, USA) were adopted for cell migration and invasion analyses. For invasion assay, the bottom of the upper chamber of Transwell plates was pre-coated with 60 μL Matrigel (diluted with serum-free medium). About 1 × 10^5^/mL SiHa and HeLa cells were inoculated into the upper chambers with 500 μL complete medium in the bottom chambers. Twenty 4 h later, cells migrated and invaded into the lower compartment were stained with crystal violet, and then counted with a microscope.

### Tube formation assay

Per well of 96-well plates was pre-coated with 50 µL chilled Matrigel for 30 min. Then about 100 µL conditioned medium (CM) of SiHa and HeLa cells with indicated transfection was collected and transferred into each well. Thereafter, HUVECs (1 × 104/well) were added into each well and incubated for 6 h. Finally, the tubular branches were photographed and manually counted.

### Macrophage polarization analysis

THP-1 cells were stimulated by 100 ng/mL phorbol 12-myristate 13-acetate (PMA; Abcam) for 24 h, and then underwent indicated transfection, 48 h later, cells were re-suspended in flow cytometry buffer and stained for 30 min at 4 °C with anti-CD11b and anti-CD206 antibody away from light. Lastly, CD11b + /CD206 + cells were analyzed on the flow cytometer and FlowJo software.

After PMA stimulation and indicated transfection, THP-1 cells were harvested and levels of IL-10 and TGF-β1 were detected by qRT-PCR as described above.

### Co-immunoprecipitation (Co-IP) assay

Cells were lysed in 500 mL co-IP buffer (50 mM Tris pH 7.5, 150 mM NaCl, 5 mM EDTA, 1–2% Nonidet P-40, and protease inhibitor cocktail), then the protein was separated on a Bolt 8%% Gel. Next, proteins were incubated with anti-USP7 or anti-IgG and 100 μL protein A + G agarose beads at 4 °C for 4 h. After washing, protein bounds were detected by western blotting.

### In vivo assay

The sh-NC or sh-USP7 was cloned into lentiviral plasmids, and then transfected into 293T cell. 48 h later, the supernatants of 293T cells were collected and lentiviral particles were obtained by ultracentrifugation. The lentiviral particles carrying sh-NC or sh-USP7 were used to infect SiHa cells in DMEM with 8 µg/mL polybrene and 10% FBS for 12 h. Finally, SiHa cells were collected, and about 2 × 10^5^ SiHa cells were subcutaneously inoculated into BALB/c nude mice (4–5 weeks old, *n* = 18, Slaike Jingda Laboratory, Hunan, China). Mice were divided into three groups: sh-NC, sh-USP7, or sh-USP7 + MTDH groups. In sh-USP7 + MTDH groups, mice were intratumorly injected with MTDH plasmids and Lipofectamine at 3 sites of the xenograft tumor every 3 days when the tumor volume reached about 100 mm^3^. Tumor volume was calculated following the formula: volume = 0.4 × length × width2. At day 28, all mice were killed, tumors were isolated for weighing, and then collected for western blotting as described above, or fixed in formalin for immunohistochemistry (IHC) analysis using the Ki67 antibody (Abcam, ab15580).

### Statistical analysis

The data were manifested as the mean ± standard deviation (SD). The normal distribution was determined by a Kolmogorov–Smirnov test, if not normally distributed, by Mann–Whitney test for comparison. One-way or two-way analysis of variance (multiple groups) and Student’s *t* test (two groups) were used for comparison in different groups as appropriate. The correlation was assessed using Pearson correlation coefficient.* P* < 0.05 was considered statistically significant.

## Results

### MTDH is highly expressed in cervical cancer

**Fig. 1:**
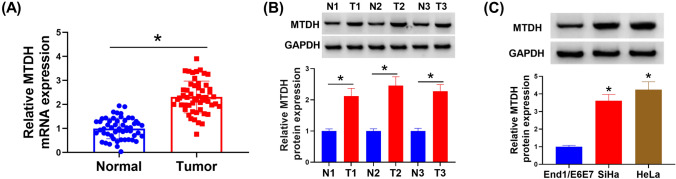
MTDH is highly expressed in cervical cancer. **A**, **B** qRT-PCR and western blotting analyses for MTDH expression in CC tissues and matched normal tissues. **C** Western blotting analysis for MTDH level in CC cell lines and normal End1/E6E7 cells. *P < 0.05

As shown in Fig. 1A and B, MTDH expression was higher in CC tissues both at mRNA and protein levels than those in normal tissues. Also, its level was increased in CC cell lines compared with the normal End1/E6E7 cells (Fig. 1C). The data suggested that abnormal expression of MTDH might be associated with CC progression.

### MTDH silencing suppresses CC cell proliferation, migration, invasion, angiogenesis and macrophage M2 polarization

**Fig. 2:**
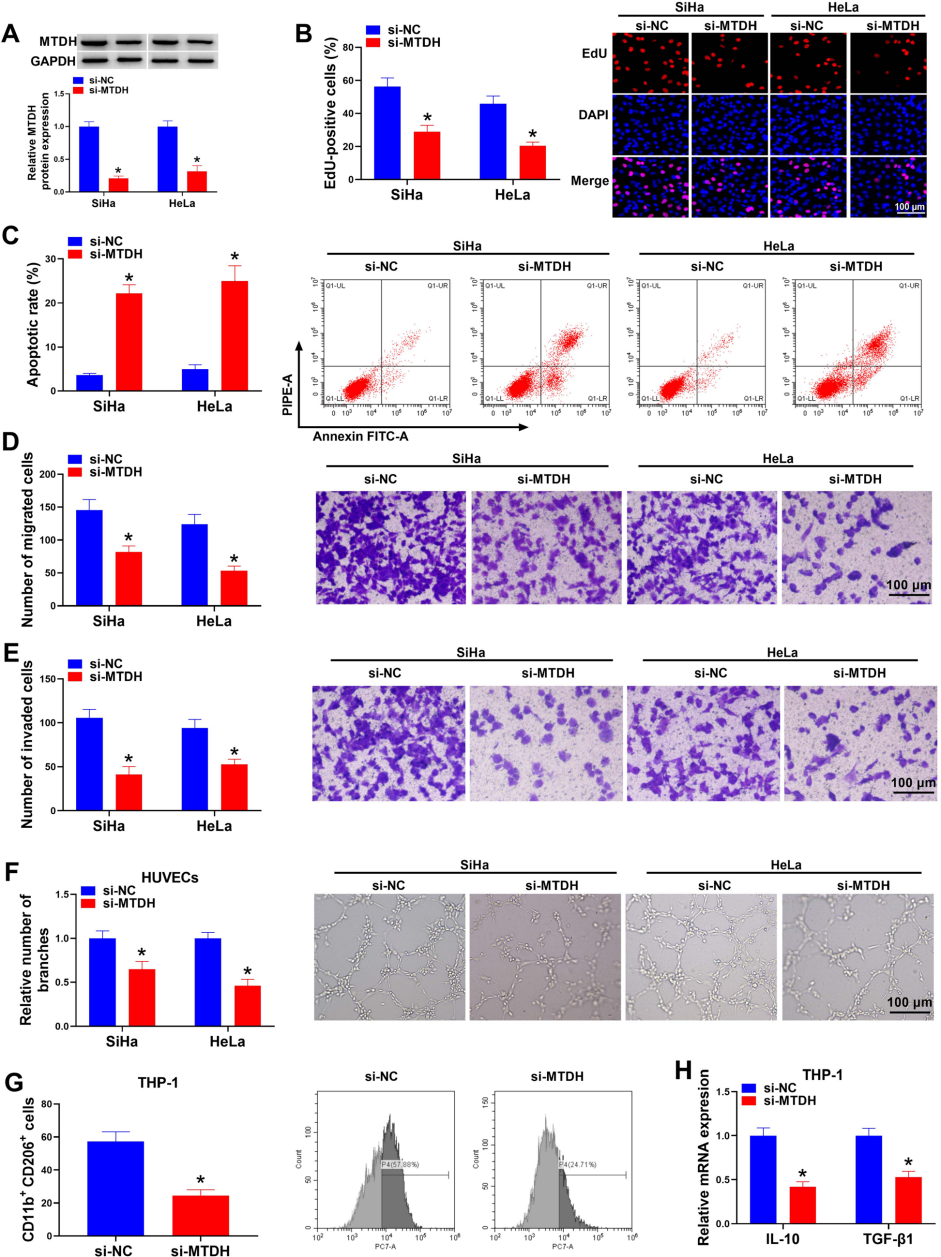
MTDH silencing suppresses CC cell proliferation, migration, invasion, angiogenesis and macrophage M2 polarization. **A**–**F** SiHa and HeLa cells were transfected with si-NC or si-MTDH. **A** Detection of MTDH levels in cells by western blotting. **B** EdU assay for cell proliferation. **C** Flow cytometry for cell apoptosis. **D**, **E** Transwell assay for cell migration and invasion abilities. **F** Tube formation assay for cell tube forming ability of HUVECs. **G**, **H** THP-1 cells were transfected with si-NC or si-MTDH. **G** Measurement of CD206 + macrophages by flow cytometry. **H** qRT-PCR analysis for IL-10 and TGF-β1 levels in THP-1 cells. *P < 0.05

Next, the action of MTDH on CC progression was determined. Western blotting analysis showed that the introduction of si-MTDH markedly reduced MTDH expression level in CC cell lines (Fig. 2A). Functionally, MTDH knockdown suppressed SiHa and HeLa cell proliferation but induced cell apoptosis (Fig. 2B, C). Transwell assay showed that the migration and invasion abilities of SiHa and HeLa cells were notably inhibited after MTDH silencing (Fig. 2D, E). Moreover, we found the tube branches formed by HUVECs were markedly reduced by MTDH deletion (Fig. 2F), implying the inhibition of cell angiogenic abilities. In addition, the decrease of CD206 + macrophages were observed after MTDH silencing (Fig. 2G), and its silencing also led to the reduction of the levels of IL-10 and TGF-β1, the markers of M2 macrophages, in THP-1 cells (Fig. 2H), suggesting that MTDH deletion suppressed macrophage M2 polarization.

### USP7 regulates MTDH protein stability through deubiquitination

**Fig. 3:**
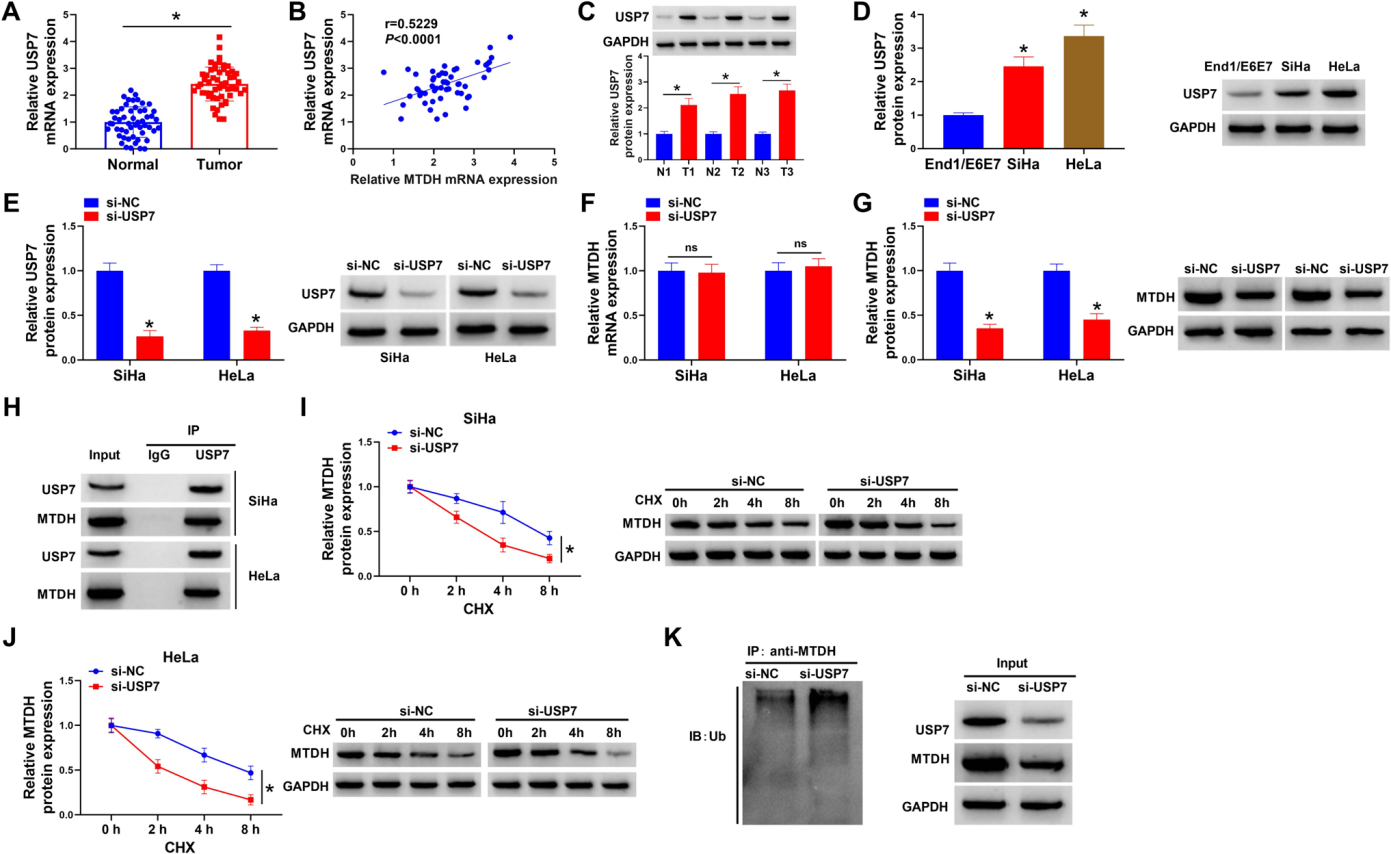
USP7 regulates MTDH protein stability through deubiquitination. **A** qRT-PCR analysis for USP7 mRNA expression in CC tissues and normal tissues. **B** USP7 expression was positively correlated with MTDH mRNA in CC tissues. **C**, **D** Western blotting for USP7 protein expression in CC tissues and matched normal tissues, as well as in CC cell lines and normal End1/E6E7 cells. **E** The interference efficiency of si-USP7 or si-NC was validated by western blotting in SiHa and HeLa cells. **F**, **G** qRT-PCR and western blotting analyses for MTDH expression in SiHa and HeLa cells after USP7 knockdown. **H** Co-IP assay for the interaction analysis between USP7 and MTDH. **I**, **J** MTDH levels were examined by western blotting in SiHa and HeLa cells transfected with si-USP7 or si-NC before CHX treatment for different time points. **K** Detection of poly-ubiquitination of MTDH after USP7 knockdown by Co-IP and western blotting analyses. *P < 0.05

As shown in Fig. 3A, USP7 mRNA was found to be up-regulated in CC tissues, moreover, its expression was positively correlated with MTDH mRNA (Fig. 3B). Also, USP7 protein expression was increased in CC tissues and cell lines (Fig. 3C, D). After confirming the interference efficiency of si-USP7 (Fig. 3E), we found USP7 silencing led to a decrease of MTDH protein expression but not the mRNA expression in CC cell lines (Fig. 3F, G), suggesting that USP7 might regulate MTDH expression at protein level. Then the interaction between USP7 and MT DH was investigated. Co-IP assay suggested that USP7 could bind to MTDH (Fig. 3H). In addition, USP7-decreased SiHa and HeLa cells were treated with cycloheximide (CHX) (a protein synthesis inhibitor) for different times to block de novo protein synthesis, it was found that the degradation of MTDH was raised by USP7 knockdown in the presence of CHX (Fig. 3I, J), suggesting that USP7 affected the stability of MTDH protein. Then we assessed whether MTDH was stabilized by USP7-mediated deubiquitination, USP7 silencing markedly erased poly-ubiquitination modification of MTDH (Fig. 3K), indicating that USP7-mediated MTDH deubiquitination.

### USP7 silencing suppresses CC cell proliferation, migration, invasion, angiogenesis and macrophage M2 polarization

**Fig. 4:**
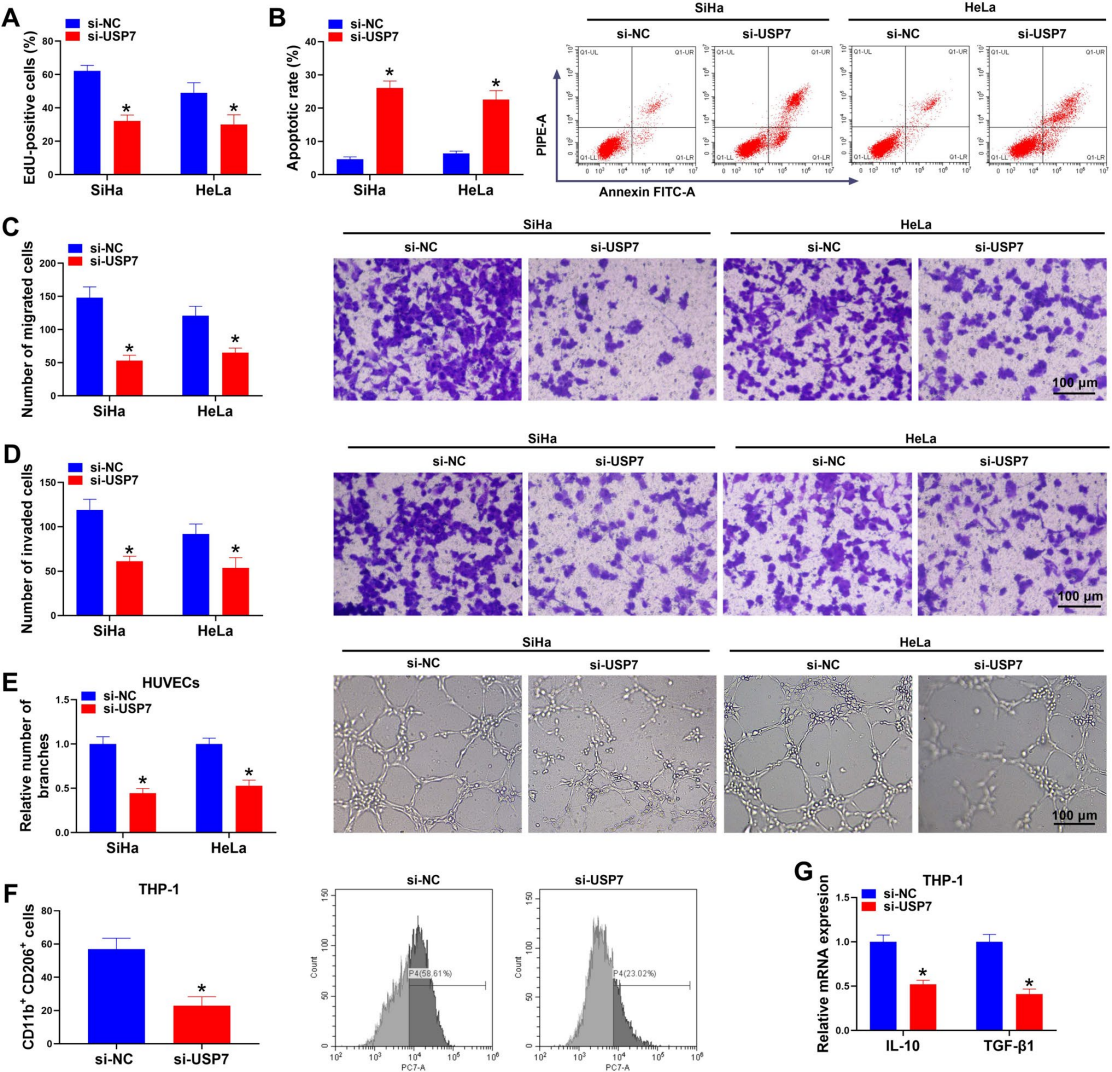
USP7 silencing suppresses CC cell proliferation, migration, invasion, angiogenesis and macrophage M2 polarization. **A**–**E** SiHa and HeLa cells were transfection with si-NC or si-USP7. **A** EdU assay for cell proliferation. **B** Flow cytometry for cell apoptosis. **C**, **D** Transwell assay for cell migration and invasion abilities. **E** Tube formation assay for cell tube forming ability of HUVECs. **F**, **G** THP-1 cells were transfected with si-NC or si-USP7. **F** Measurement of CD206 + macrophages by flow cytometry. **G** qRT-PCR analysis for IL-10 and TGF-β1 levels in THP-1 cells. *P < 0.05

Subsequently, we probed the functions of USP7 on CC progression. SiHa and HeLa cells were transfection with si-NC or si-USP7. Then it was confirmed that USP7 knockdown suppressed proliferation (Fig. 4A) and induced apoptosis (Fig. 4B) in SiHa and HeLa cells. The migratory and invasive SiHa and HeLa cells were also reduced after USP7 deletion (Fig. 4C, D). Moreover, si-USP7 introduction markedly inhibited the tube formation of HUVECs (Fig. 4E). In addition, we also found CD206 + macrophages were reduced after USP7 knockdown (Fig. 4F). And the expression levels of IL-10 and TGF-β1 were decreased by si-USP7 introduction in THP-1 cells (Fig. 4G), further indicating USP7 knockdown suppressed macrophage M2 polarization.

### USP7 regulates CC cell proliferation, migration, invasion, angiogenesis and macrophage M2 polarization by MTDH

**Fig. 5:**
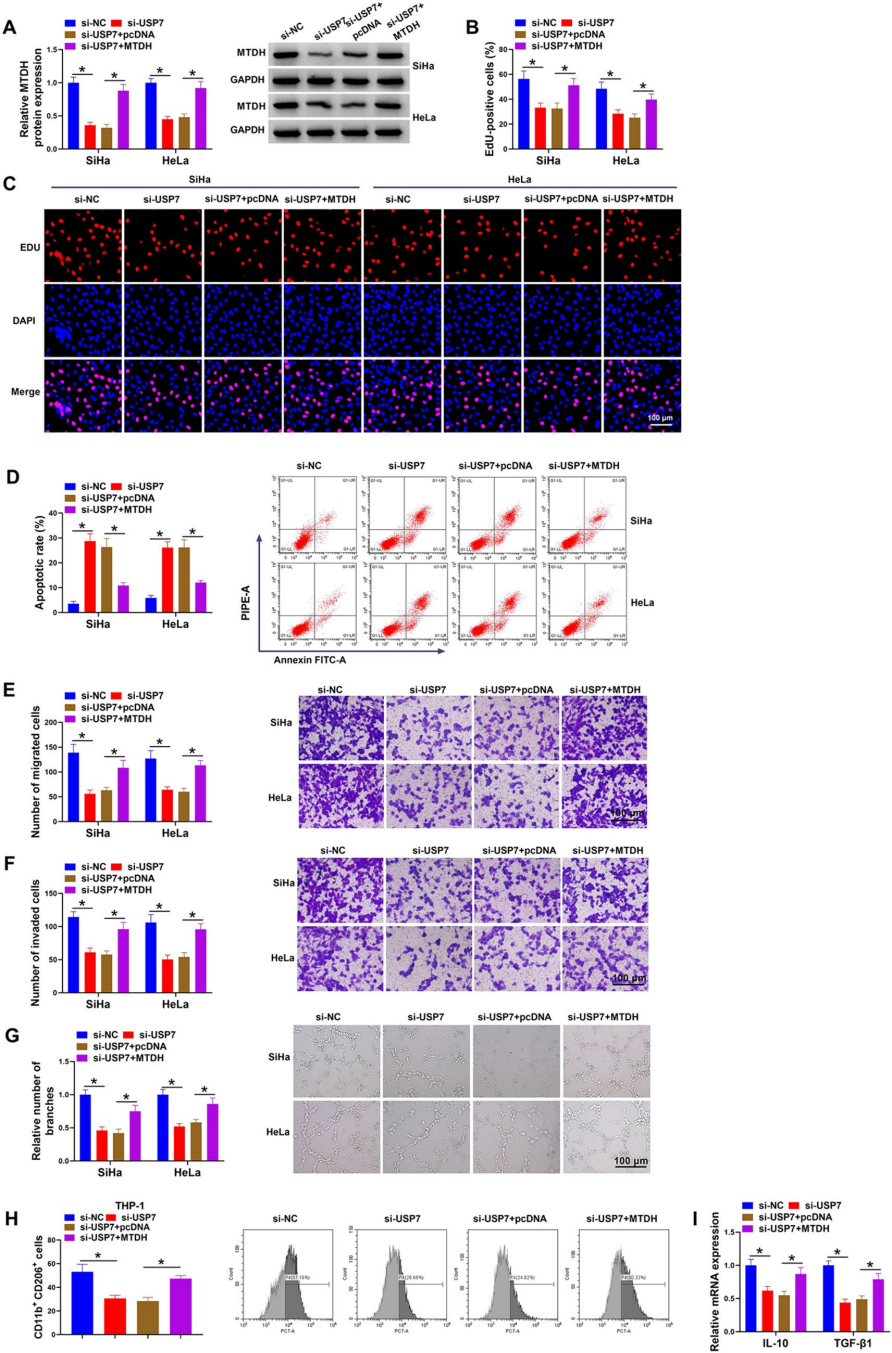
USP7 regulates CC cell proliferation, migration, invasion, angiogenesis and macrophage M2 polarization by MTDH. **A**–**F** SiHa and HeLa cells were transfection with si-USP7 alone or si-USP7 and MTDH. **A** Measurement of MTDH protein in cells by western blotting. **B** EdU assay for cell proliferation. **C** Flow cytometry for cell apoptosis. **D**, **E** Transwell assay for cell migration and invasion abilities. **F** Tube formation assay for cell tube forming ability of HUVECs. **G**, **H** THP-1 cells were transfected with si-USP7 alone or si-USP7 and MTDH. **G** Measurement of CD206 + macrophages by flow cytometry. **H** qRT-PCR analysis for IL-10 and TGF-β1 levels in THP-1 cells. *P < 0.05

Thereafter, USP7 affected CC progression via MTDH was explored. SiHa and HeLa cells were transfection with si-USP7 alone or si-USP7 and MTDH. Western blotting analysis showed that MTDH vector introduction rescued si-USP7-induced decrease of MTDH level in cells (Fig. 5A). Further functional analysis showed that the inhibition of cell proliferation (Fig. 5B), promotion of cell apoptosis (Fig. 5C), reduction of the number of migratory and invasive cells (Fig. 5D, E) caused by si-USP7 were reversed by MTDH up-regulation in SiHa and HeLa cells. The reduction of HUVEC-formed tubular structures mediated by si-USP7 were also abolished by MTDH up-regulation (Fig. 5F). Moreover, MTDH overexpression rescued si-USP7-induced attest of macrophage M2 polarization, evidenced by increased CD206 + macrophages, and levels of IL-10 and TGF-β1 in THP-1 cells (Fig. 5G, H).

### USP7 silencing impedes CC growth by regulating MTDH in vivo

**Fig. 6:**
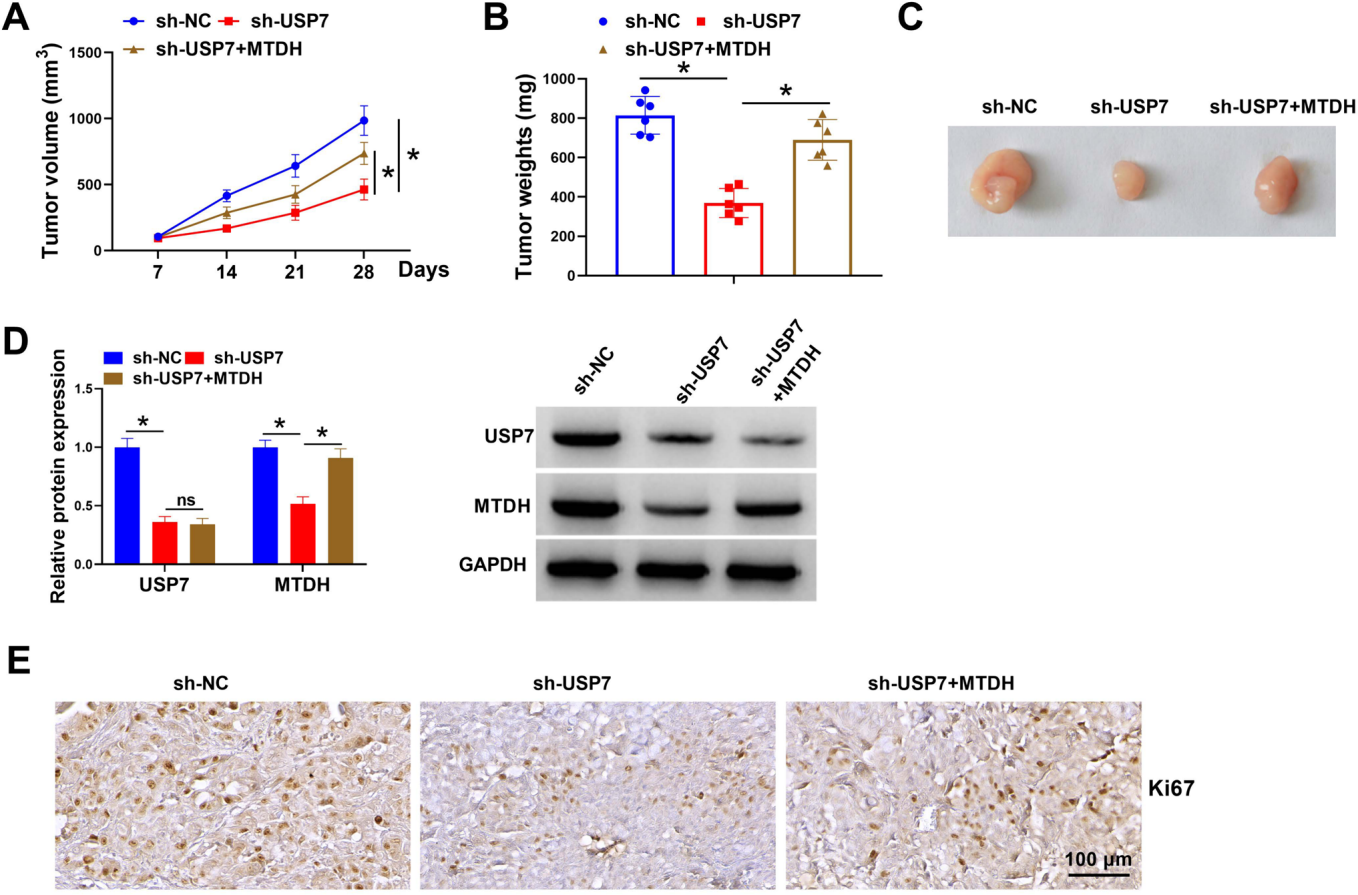
USP7 silencing impedes CC growth by regulating MTDH in vivo. **A** The growth curve of xenograft tumors, **B** the weight of xenograft tumors and **C** representative xenografts. **D** Detection of USP7 and MTDH levels in xenograft tumors by western blotting. **E** IHC analysis for Ki67 protein in xenograft tumors. *P < 0.05

To explore the action of USP7 and MTDH in tumor growth in vivo, a mouse xenograft model was established. As exhibited in Fig. 6A–C, USP7 knockdown suppressed tumor growth in vivo, reflected by smaller tumor volume and lighter tumor weight in sh-USP7 group, while MTDH overexpression reversed the anti-growth effect of sh-USP7. Western blotting showed that USP7 and MTDH levels were decreased in tumors of sh-USP7 group, and MTDH level but not USP7 was rescued in tumors of sh-USP7 + MTDH group (Fig. 6D). IHC analysis further showed that Ki67 protein, a marker of proliferation, was reduced in tumors of sh-USP7 group, but elevated in tumors of sh-USP7 + MTDH group (Fig. 6E).

## Discussion

MTDH deregulation was related to many of human cancers, and proliferation, metastasis, chemoresistance, and angiogenesis were included in MTDH-induced malignant hallmarks in cancer cells (Wang et al. 2019; Shen et al. 2021; Sarkar 2013). In our work, we found an increased expression of MTDH in CC tissues and cell lines. Functionally, MTDH silencing suppressed CC cell proliferation, migration, invasion, and angiogenesis. Moreover, we also found MTDH deletion suppressed macrophage M2 polarization. Macrophages are innate immune cells that are pivotal for the removal of superfluous cells, tissue homeostasis, and immune responses to infections, they are usually classified into M1 and M2 macrophages, in which M2 macrophages have higher phagocytic activity and secrete TGF-β, IL-10, and VEGF, showing anti-inflammatory activity and promoting neoangiogenesis, tissue remodeling, and cancer progression (Italiani and Boraschi 2014; Cendrowicz et al. 2021). Thus, in addition to the promoting action of MTDH on CC cell proliferation, migration, invasiveness, and angiogenesis, we also confirmed that MTDH could suppress macrophage M2 polarization to promote immune suppression in CC cells.

For further mechanism analysis, we confirmed that USP7 directly interacted with MTDH and stabilized its expression via deubiquitination. USP7, a most widely studied deubiquitination enzyme yet, is identified to perform oncogenic activity to facilitate cancer growth and is detrimental to the immune response to cancer (Wang et al. 2016; Everett 2014). USP7 has many reported substrates (≥ 20), and one possibility mechanism of USP7 plays a key role in affecting cancer progression is that USP7 function as a binding protein to regulate the expression and stabilities of genes (N-Myc, PTEN, PCNA, FOXO4, p53 and so on) associated with tumorigenesis and proliferation (Zhou et al. 2018; Yeasmin Khusbu et al. 2018; Schauer et al. 2020). Dai et al*.* showed that USP7 deletion accelerated the tumor infiltration of M1 macrophages and favored IFN-γ + CD8 + T cell proliferation, and then impeded lung cancer growth (Dai et al. 2020). USP7 deubiquitinated PLK1 to sustain its protein stability, and then promoted cell proliferation, taxane resistance, and chromosome misalignment in prostate cancer (Peng et al. 2019). Yu et al*.* discovered that A11 could reverse USP7-mediated PD-L1 deubiquitination, then degraded PD-L1 to repress immune evasion and enhance the killing effects of T cells in multiple tumors (Yu et al. 2023). In our study, we observed an increased-USP7 expression in CC. The silencing of USP7 could impair CC cell proliferation, migration, invasion, and angiogenesis, as well as suppress macrophage M2 polarization. Moreover, the anticancer effects mediated by USP7 silencing were rescued by MTDH overexpression. Importantly, we also confirmed that USP7 deletion hindered CC growth in mice by regulating MTDH.

In conclusion, USP7 deubiquitinated MTDH to stabilize its expression, and then contributed to CC cell proliferation, migration, invasion, and angiogenesis, as well as macrophage M2 polarization in vitro, and enhanced tumor growth in nude mice. Nevertheless, the role of USP7 and MTDH in the tumor microenvironment still needs further investigate in immune-competent mouse model to identify their effects in immunoregulation. Even so, this research also provide a promising new idea for developing effective molecular therapy for CC patients.

## Data Availability

The analyzed data sets generated during the present study are available from the corresponding author on reasonable request.
